# From the root to the stem: interaction between the biocontrol root endophyte *Pseudomonas fluorescens* PICF7 and the pathogen *Pseudomonas savastanoi* NCPPB 3335 in olive knots

**DOI:** 10.1111/1751-7915.12036

**Published:** 2013-02-20

**Authors:** M Mercedes Maldonado-González, Pilar Prieto, Cayo Ramos, Jesús Mercado-Blanco

**Affiliations:** 1 Departamento de Protección de Cultivos, Instituto de Agricultura Sostenible, Consejo Superior de Investigaciones Científicas (CSIC)Alameda del Obispo s/n, Apartado 4084, E-14080, Córdoba, Spain; 2 Departamento de Mejora Genética Vegetal, Instituto de Agricultura Sostenible, Consejo Superior de Investigaciones Científicas (CSIC)Alameda del Obispo s/n, Apartado 4084, E-14080, Córdoba, Spain; 3 Instituto de Hortofruticultura Subtropical y Mediterránea ‘La Mayora’, Universidad de Málaga-Consejo Superior de Investigaciones Científicas (IHSM-UMA-CSIC), Área de Genética, Facultad de Ciencias, Universidad de MálagaCampus de Teatinos s/n, E-29071, Málaga, Spain

## Abstract

Olive knot disease, caused by *Pseudomonas savastanoi* pv. savastanoi, is one of the most important biotic constraints for olive cultivation. *Pseudomonas fluorescens* PICF7, a natural colonizer of olive roots and effective biological control agent (BCA) against Verticillium wilt of olive, was examined as potential BCA against olive knot disease. Bioassays using *in vitro*-propagated olive plants were carried out to assess whether strain PICF7 controlled knot development either when co-inoculated with the pathogen in stems or when the BCA (in roots) and the pathogen (in stems) were spatially separated. Results showed that PICF7 was able to establish and persist in stem tissues upon artificial inoculation. While PICF7 was not able to suppress disease development, its presence transiently decreased pathogen population size, produced less necrotic tumours, and sharply altered the localization of the pathogen in the hyperplasic tissue, which may pose epidemiological consequences. Confocal laser scanning microscopy combined with fluorescent tagging of bacteria revealed that when PICF7 was absent the pathogen tended to be localized at the knot surface. However, presence of the BCA seemed to confine *P. savastanoi* at inner regions of the tumours. This approach has also enabled to prove that the pathogen can moved systemically beyond the hypertrophied tissue.

## Introduction

*Pseudomonas savastanoi* pv. savastanoi (Psv) (Gardan *et al*., 1992; Sisto *et al*., 1999) is the causal agent of olive (*Olea europaea* L.) knot disease (Kennelly *et al*., 2007; Ramos *et al*., 2012) and an unorthodox member of the *Pseudomonas syringae* complex, encompassing at least 60 pathovars and several other *Pseudomonas* species (Gardan *et al*., 1999; Bull *et al*., 2010; Young, 2010). Infection of olive by Psv results in overgrowth formation (tumours, galls or knots) on the stems and branches of the host plant and, occasionally, on leaves and fruits. Olive knot is worldwide distributed and it is considered one of the most important diseases of this woody crop. Although losses caused by olive knot are difficult to assess and greatly depend on geographical location and olive cultivar, tree vigour, growth and yield have been reported to be moderately or severely reduced in infected trees, as well as the size and quality of fruits (Schroth *et al*., 1973; Young, 2004; Quesada *et al*., 2010a). The pathogen does not survive for long in soil, and is normally found as an epiphyte and also endophytically (Ercolani, 1978; Penyalver *et al*., 2006; Quesada *et al*., 2007), being able to move over short distances within olive orchards through dissemination of epiphytic bacteria (Quesada *et al*., 2010a). Natural isolates of Psv are phenotypically and genotypically heterogeneous, exhibiting broad virulence diversity (Penyalver *et al*., 2006) as well as variation in size and morphology of induced tumours (Pérez-Martínez *et al*., 2007). Psv NCPPB 3335, a highly virulent strain both in adult trees (Pérez-Martínez *et al*., 2007) and in micropropagated olive plants (Rodríguez-Moreno *et al*., 2008; 2009), is being used as a model organism for the study of the molecular basis of the disease onset and development (tumour formation) in woody hosts. The draft genome sequence of NCPPB 3335 (Rodríguez-Palenzuela *et al*., 2010) and the closed sequence of its three native plasmids (Bardaji *et al*., 2011) have been recently obtained.

Olive knot cannot be eradicated once established in plants, and its control must therefore be based on preventive measures (Young, 2004; Quesada *et al*., 2010a,b; Ramos *et al*., 2012). However, from an integrated disease management strategy perspective only a few control measures have proved to be effective. For instance, olive cultivars completely resistant to the pathogen are not yet available (Penyalver *et al*., 2006). Thus, chemical control involving regular application of copper compounds has been traditionally used to manage the disease (Teviotdale and Krueger, 2004; Young, 2004; Quesada *et al*., 2010b), posing environmental risks and enhancing the likelihood of pathogen resistance. Regarding to biological control of olive knot disease, antagonistic bacteria against Psv have been isolated, comprising a bacteriocin-producing *P. syringae* pv. ciccaronei strain (Lavermicocca *et al*., 2002), a collection of fluorescent *Pseudomonas* strains isolated from the rhizosphere of different plants (Rokni Zadeh *et al*., 2008), including a *Pseudomonas putida* isolate producing a salicylate-containing antibiotic (Vlassak *et al*., 1992; Li *et al*., 2011), and several *P. fluorescens* and *Bacillus subtilis* strains isolated from olive knots and from leaves of Psv-infected trees (Krid *et al*., 2010). Although some of these strains have shown to reduce olive knot symptoms (Lavermicocca *et al*., 2002; Krid *et al*., 2012), little is known about the *in planta* community interplay between these antagonistic bacteria and the pathogen in the development of the disease.

*Pseudomonas fluorescens* PICF7 is a natural inhabitant of the olive rhizosphere isolated from roots of nursery-propagated olive plants (cv. Picual) (Mercado-Blanco *et al*., 2004). This strain has been shown to be an effective biological control agent (BCA) against Verticillium wilt of olive (Mercado-Blanco *et al*., 2004; Prieto *et al*., 2009), a disease caused by the soil-borne fungal pathogen *Verticillium dahliae* Kleb., and currently considered one of the most important biotic constraints for this woody crop (López-Escudero and Mercado-Blanco, 2011). Strain PICF7 has also been shown to develop an endophytic lifestyle within olive root tissues under diverse experimental conditions (Prieto and Mercado-Blanco, 2008; Prieto *et al*., 2009; 2011). Recent functional genomics analysis has revealed that root colonization by PICF7 induces a broad range of defence responses in olive root tissues as well as the activation of diverse transcription factors known to be involved in systemic defence responses (Schilirò *et al*., 2012). This depicts a scenario where PICF7 might be an effective BCA against other pathogens infecting olive, although additional biocontrol mechanisms (i.e. antibiosis) deployed by PICF7 and operating *in planta* cannot be completely ruled out.

In this study we evaluate the potential of *P. fluorescens* PICF7 to be used as a BCA against olive knot disease both in *in vitro*-propagated explants and in lignified, pot-acclimated plants. We tested the hypothesis whether PICF7, a natural inhabitant of olive roots, can be effective against a pathogen which affects above-ground organs of the same host under two different situations: (i) the BCA applied to the roots (its natural niche) and the pathogen inoculated into the stems, and (ii) both microorganisms co-inoculated in artificially produced wounds on the favourable, natural environment of the pathogen (stems). We assessed strain PICF7's ability to: (i) colonize and persist in olive stem tissues; (ii) influence the establishment of the pathogen on/in its target niche; and (iii) affect olive knot development. The interaction between *P. fluorescens* PICF7 and Psv NCPPB 3335 was investigated *in planta* at both macro- and microscopic levels.

## Results

### *Pseudomonas fluorescens* PICF7 antagonizes *Pseudomonas savastanoi* NCPPB 3335 *in vitro* and colonizes roots and stems of *in vitro*-propagated olive plants

In order to assess whether the indigenous, olive roots inhabitant *P. fluorescens* PICF7 has potential as a BCA against Psv, its effectiveness to antagonize the pathogen *in vitro* and to colonize stems (and roots) of *in vitro*-propagated olive plants were first evaluated. *In vitro* antagonism assays using different culturing media [Potato Dextrosa Agar (PDA), King's B Agar (KBA), and Luria–Bertani Agar (LBA)] showed that strain PICF7 strongly inhibited the growth of Psv NCPPB 3335 in PDA plates (Fig. S1). However, inhibition haloes in KBA and LBA media were negligible or restricted to the very proximal region surrounding the BCA colony. The relative size of growth inhibition haloes on PDA plates varied depending on the concentration of Psv and on the incubation temperature. For instance, PDA plates harbouring 10^5^ cfu (colony-forming units) ml^−1^ of Psv yielded inhibition haloes with average relative sizes of 0.8 ± 0.03 (at 25°C) and 0.8 ± 0.02 (at 28°C). On the contrary, when the pathogen population on PDA plates increased up to 10^8^ cfu ml^−1^, relative size of inhibition haloes were 0.6 ± 0.03 (at 25°C) and 0.4 ± 0.08 (at 28°C).

The ability of *P. fluorescens* PICF7 to colonize roots and aboveground tissues of *in vitro-*propagated olive explants was confirmed as well. When the BCA was applied to the root system, colony counts from roots macerates showed that population size of strain PICF7 was stably maintained along the experiment [8.8 ± 0.4 (mean log_10_ cfu g^−1^ fresh root/stem tissues ± SD) at 0 days after inoculation (DAI) and 8 ± 1.4 at 60 DAI]. Moreover, GFP-tagged PICF7 colonized endophytically root tissues, and root hairs were found to be important is this process (data not shown). On the contrary, when the BCA was inoculated in the stems, population size of PICF7 increased over time in the segment containing the inoculation point (3.2 ± 0.4 and 5.9 ± 0.6 at 0 and 60 DAI respectively). On the other hand, the possible translocation of PICF7 cells from roots to stems was also examined. Results showed that movement of strain PICF7 from inoculated roots to stems was not evident (12 out of 20 plants examined) or, at most, it remained restricted to the basal segment of the stems (six plants) and with a highly variable population scored (2.4–7.3 at 50 DAI). Only in two plants PICF7 cells were detected in the upper segment of the stems (4.1 and 6 at 50 DAI). Nevertheless, cross-contamination during plant manipulation could not be completely ruled out for these cases. No bacteria were detected in roots and stems of non-inoculated (control) plants at any time.

### *Pseudomonas fluorescens* PICF7 applied to roots does not suppress olive knot development

Bioassays designed to assess the ability of *P. fluorescens* PICF7 to control the onset and development of olive knot disease by means of systemic defence response(s) showed that PICF7 was not able to suppress the disease under the experimental conditions used despite its ability to colonize the root system. Thus, when the BCA and the pathogen were spatially separated (roots of *in vitro-*propagated olive explants bacterized with PICF7 1 week prior to Psv inoculation in stems), development of knots and their anatomy did not differ regardless the presence or absence of PICF7 (data not shown). Moreover, population sizes of strain NCPPB 3335 scored in hyperplasic tissues of PICF7-bacterized (3.1 ± 0.5 at 0 DAI and 6.5 ± 1.2 at 60 DAI) and control (3.8 ± 0.6 at 0 DAI and 7.1 ± 0.2 at 60 DAI) plants were not significantly (*P* > 0.05) different along the experiment.

### Presence of *Pseudomonas fluorescens* PICF7 in stems affects pathogen population and knot development

**Figure 1:**
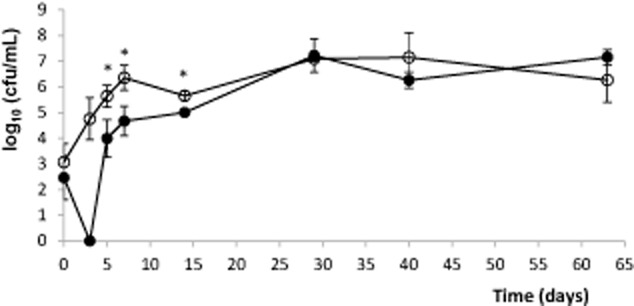
Population size of *Pseudomonas savastanoi* NCPPB 3335 recovered from inoculation sites or developed knots from a 63 days' bioassay performed with *in vitro-*propagated olive plants co-inoculated (•) or not (○) with *Pseudomonas fluorescens* PICF7 (see text for details). Each score time-point is the mean from three independent samples. Error bars represent standard deviation. Mean values significantly different (*P* < 0.05) according to Student's *t*-test are marked by asterisks. Results shown are from a representative bioassay. This experiment was repeated three times with similar results.

**Figure 2:**
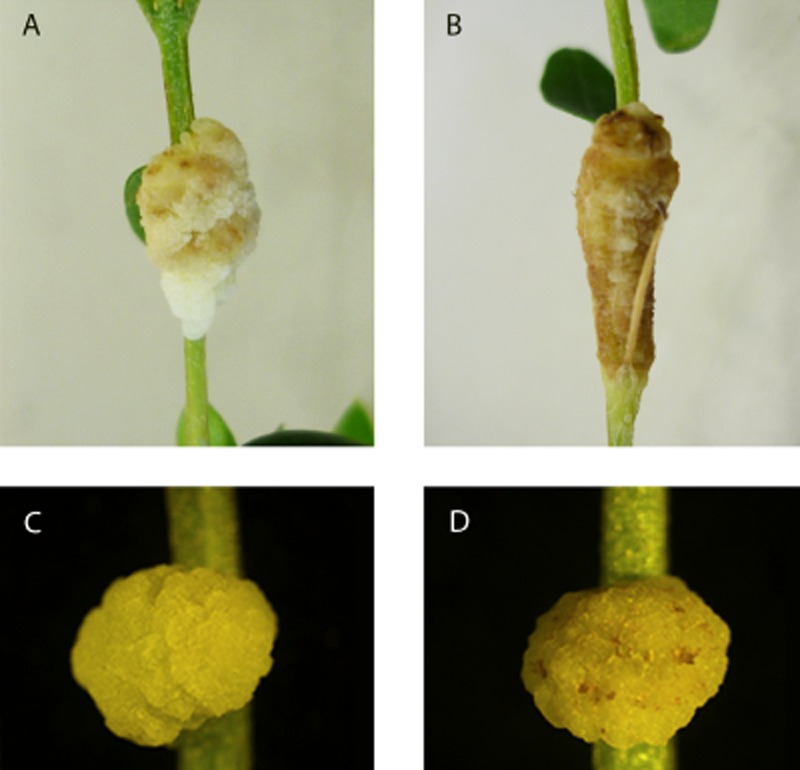
Tumours produced on *in vitro-*propagated olive plants by *Pseudomonas savastanoi* NCPPB 3335 (Psv) in the presence (A) or in the absence (B) of *Pseudomonas fluorescens* PICF7 at 58 DAI. Tumours developed in a different experiment upon inoculation with Psv-GFP in the presence (C) and in the absence (D) of PICF7 were also observed at 27 DAI. Presence of PICF7 in the inoculation mix produced tumours with reduced or no necrosis (A and C) regardless the presence of Psv or its GFP-tagged derivative (see text for details). Panels B and D show tumours with symptoms of necrosis.

Results showed that when *P. fluorescens* PICF7 was inoculated into the stems along with the pathogen, population size of NCPPB 3335 sharply decreased and was significantly (*P* < 0.05) lower during the first 2 weeks after bacterization, compare with that scored in plants only inoculated with Psv (control treatment). However, this fall in the pathogen population was only transitory and Psv counts recovered later on and did not differ significantly (*P* > 0.05) between treatments until the end of the bioassays (64 DAI) (Fig. 1). Population sizes of PICF7 did not significantly differ (*P* > 0.05) regardless the presence (3.3 ± 0.2 at 0 DAI and 6.4 ± 0.4 at 60 DAI around the inoculation points and developed tumours respectively) or absence (segments containing the inoculation point, see above) of the pathogen. Interestingly enough, co-inoculation of PICF7 with Psv significantly altered the macroscopic appearance of the tumours. Thus, less necrotic knots (discoloured, whitish tumours) developed when PICF7 was co-inoculated with the pathogen (Fig. 2A and C) in comparison with knots developed in plants inoculated with Psv alone (Fig. 2B and D).

**Figure 3:**
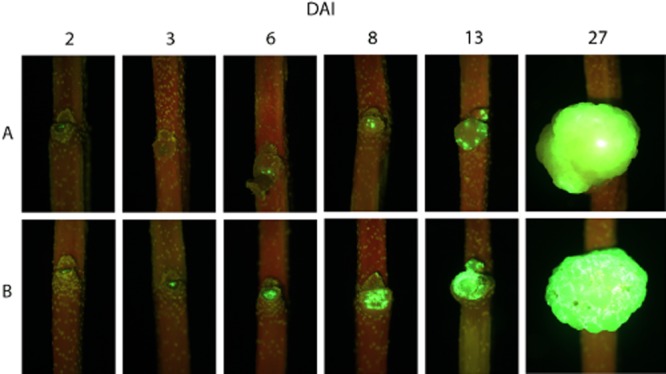
Epifluorescence microscopy images showing the presence of GFP-tagged *Pseudomonas savastanoi* NCPPB 3335 (Psv) at the inoculation point and tumours developed on *in vitro*-propagated olive plants during a time-course experiment (27 days). Stems were inoculated with the pathogen alone (B) or mixed with *Pseudomonas fluorescens* PICF7 (A) (see text for details). Two plants were analysed per each sampling time-point with similar results. Green fluorescence reveals the presence of living Psv-GFP cells. Plants co-inoculated with Psv-GFP and PICF7 exhibited no detectable fluorescence (3 DAI) or less fluorescent areas (6, 8, 13 DAI) compared with plants inoculated with Psv-GFP alone. At the end of the experiment (27 DAI), tumours differ neither in size nor in fluorescence between treatments.

To further check that transient decrease of Psv population and modification of tumour's macroscopic appearance were due to the presence of strain PICF7, epifluorescence microscopy combined with fluorescent tagging of Psv NCPPB 3335 with GFP (Psv-GFP) was used. In agreement with results from previous bioassays, a decrease of Psv NCPPB 3335 population in tumours developed in plants co-inoculated with the BCA was visualized during the first 14 days. Lower population of the pathogen was revealed as a depletion of the green fluorescence at the inoculation points and within the hyperplasic tissue developed in Psv/PICF7 co-inoculated plants (Fig. 3A, 2–13 DAI), compared with those ones only inoculated with Psv alone (Fig. 3B, 2–13 DAI). As in previous bioassays, NCPPB 3335 population in Psv/PICF7 co-inoculated plants recovered over time, and tumours from both treatments reached similar levels of fluorescence (Fig. 3A, 27 DAI and B, 27 DAI). Population size of Psv recovered from knots along the experiment confirmed that the observed fluorescence fluctuation correlated to a decrease in Psv colony counts (approximately 1.5 order of magnitude lower) at all times except at 27 DAI (6.8 ± 0.3, for Psv alone and 6.5 ± 0.1, for Psv/PICF7 coinoculated plants), as shown in previous bioassays (see above). Finally, macroscopic appearance of tumours developed in this assay differed depending on the presence or not of the BCA (Fig. 2C and D), confirming previous observations.

### Co-inoculation of *Pseudomonas fluorescens* PICF7 alters the localization and distribution of Psv in tumours

**Figure 4:**
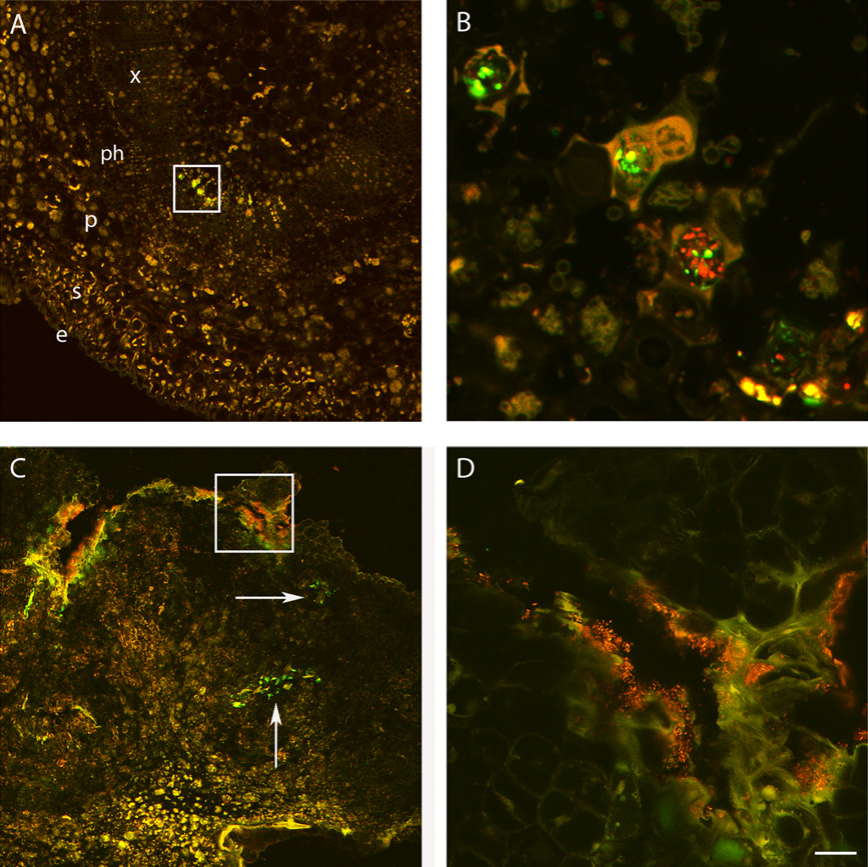
Confocal laser scanning microscopy images of transversal vibratome tumour sections (40 μm thick) showing localization of *Pseudomonas savastanoi* (Psv-GFP, green) and *Pseudomonas fluorescens* (PICF7-RFP, red). Images were taken at 1 (A, B) and 4 (C, D) weeks after inoculation with Psv-GFP and PICF7-RFP. A. Vibratome transversal section of a representative stem 1 week after inoculation. Fluorescence located inside xylem vessels is due to the presence of intermixed Psv-GFP and PICF7-RFP cells (inset). B. Inset in (A) showing Psv-GFP and PICF7-RFP cells intermixed inside the vascular vessel cells. C. Tumour sampled 4 weeks after inoculation showing events of inner (arrowed) and surface (inset) localization of Psv-GFP and PICF7-RFP cells respectively. D. Inset in (C) showing PICF7-RFP cells at the surface of the knot and a small individual colony of Psv-GFP cells in a different focus plane and not mixed with PICF7-RFP cells (visible as weak green fluorescence at the bottom of the panel). Scale bar represents 100 μm in A, 20 μm in B, 150 μm in C and 25 μm in D. e, epidermis; s, sclereids; p, parenchyma; ph, phloem; x, xylem.

**Figure 5:**
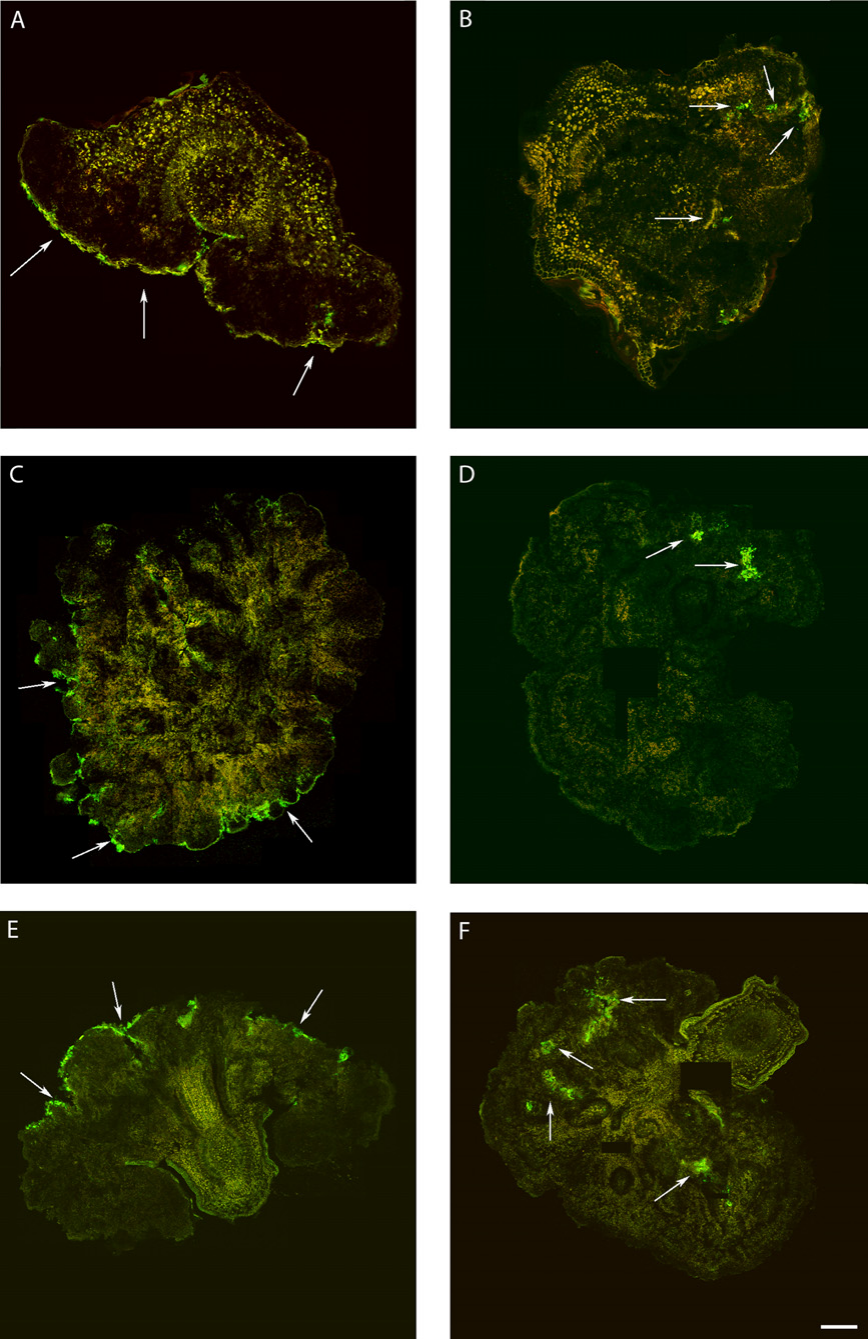
Confocal laser scanning microscopy images showing the time-course of colonization of *in vitro*-propagated olive tissues by GFP-tagged *Pseudomonas savastanoi* NCPPB3335 (Psv-GFP) in the absence (A, C and E) or in the presence (B, D, F) of *Pseudomonas fluorescens* PICF7. Transversal vibratome tumour sections (40 μm thick) were made to show inner colonization. Each panel is a composition of several images to show the whole knot and from two different bioassays. In the absence of *P. fluorescens* PICF7, Psv-GFP is visualized predominantly and profusely at the knots surface (arrowed) at 2 (A), 6 (C) and 9 (E) weeks after inoculation. In the presence of *Pseudomonas fluorescens* PICF7, Psv-GFP is visualized in inner cavities of the tumour (arrowed) at 2 (B), 7 (D) and 9 (F) weeks after inoculation. Scale bar represents 500 μm in all panels except in (A and B) where it represents 125 μm.

To assess whether differences observed in the external, macroscopic anatomy between tumours developed in Psv-inoculated and Psv/PICF7 co-inoculated plants could correlate to changes in pathogen distribution mediated by PICF7 presence, fluorescent tagging of bacteria (Psv-GFP and PICF7-RFP), vibratome-sectioning of knot and stem tissues and CLSM were used. By combining these microscopy and biotechnological tools we aimed to explore the inner anatomy of tumours as well as the localization and distribution of the BCA and the pathogen on and within knots *in vivo*, without implementing further tissue manipulation, fixation and/or staining procedures. Overall, sectioning of knots from plants co-inoculated with Psv and PICF7 was more difficult, as they presented spongy consistency compared with tumours generated by single inoculation of Psv. CLSM images showed that, in co-inoculated plants, both bacteria could be found mixed within vascular vessels of the stem at early stages of the knot development (7 DAI, Fig. 4A and B). However, from 2 weeks after artificial inoculation of bacteria until the end of the experiments (6–9 weeks), each bacterial species tended to be allocated in different regions of the tumours in most of the observations (Fig. 4C). Indeed, while both fluorescently tagged *Pseudomonas* could be found mixed at any place within the knot, particularly at the beginning of the knot development (Fig. 4A and B), PICF7-RFP cells were predominantly visualized at the knot surface or in outer regions of the tumour (Fig. 4C and D). In contrast, Psv-GFP cells were mainly found at the inner regions of the hyperplasic tissue, particularly at later times of the experiment (Fig. 4C; Fig. 5B, D and F). Remarkably, localization of Psv colonies greatly differed depending on the presence of strain PICF7. Results showed that when strain NCPPB 3335 was inoculated alone the pathogen predominantly colonized the surface of the tumour (Fig. 5A, C and E).

### Systemic movement of *Pseudomonas savastanoi* NCPPB 3335 along olive stems

**Figure 6:**
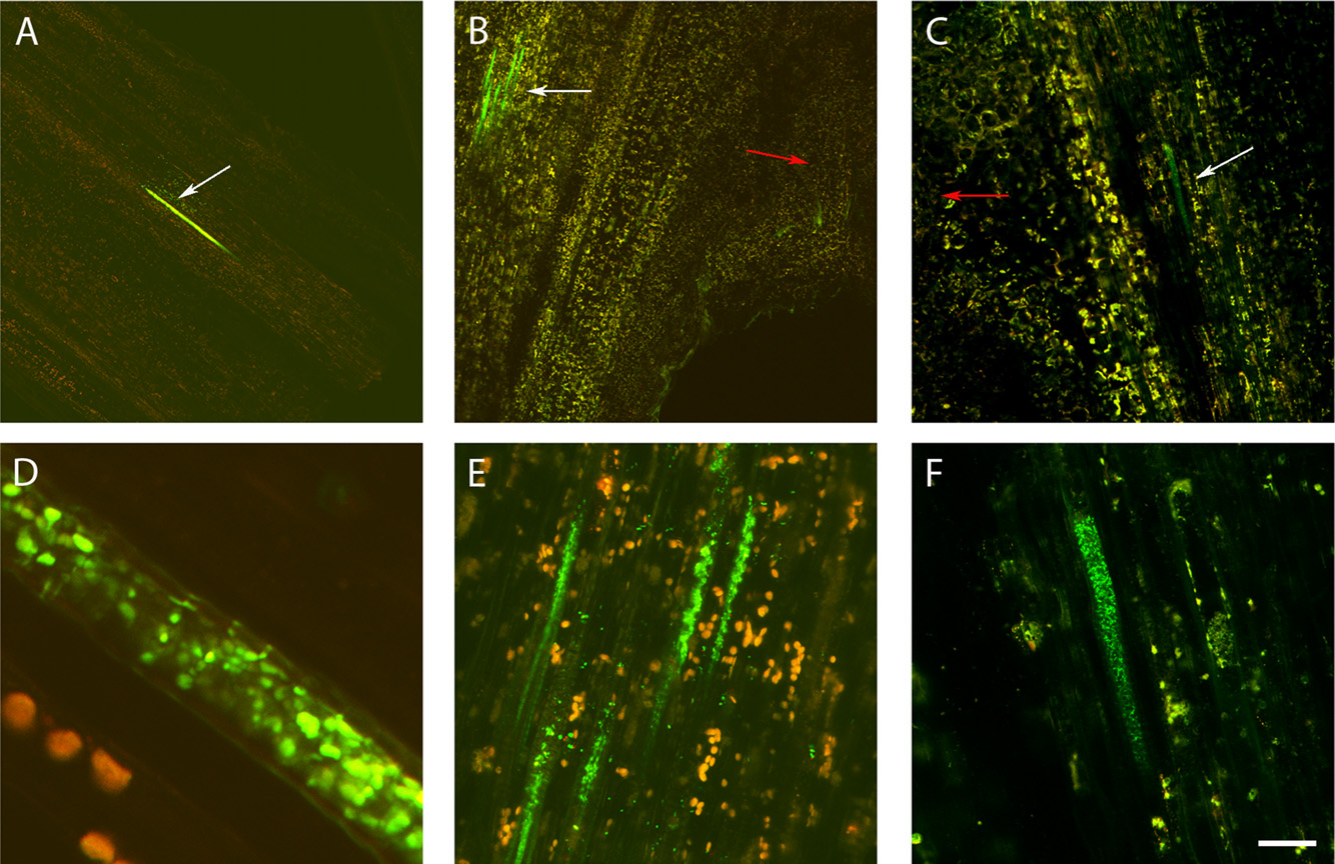
Confocal laser scanning microscopy images of *in vitro*-propagated olive plants showing the translocation of GFP-tagged *Pseudomonas savastanoi* NCPPB 3335 (Psv-GFP) from the hyperplasic tissue to the olive stems. Vibratome longitudinal sections of stems (40 μm thick) were made to show Psv-GFP internal colonization of olive vascular vessels (white arrows) away from the knot (A) and close to it (B, tumour marked by a red arrow) 6 weeks after pathogen inoculation. C. Presence of Psv-GFP in olive vascular vessels (white arrow) by the knot (red arrow) in a plant co-inoculated with *Pseudomonas fluorescens* PICF7. D, E and F are magnifications of A, B and C, respectively, showing details of olive stem vascular vessels profusely colonized by the fluorescently tagged pathogen. Scale bar represents 200 μm in A and B, 65 μm in C, 5 μm in D and 20 μm in E and F.

An interesting finding from CLSM experiments was the repeated observation of Psv-GFP colonies in stem tissues outside the hyperplasic region. Thus, CLSM imagery revealed that the pathogen could move from the inoculation point to healthy areas of the stem, colonizing the xylem vessels (Fig. 6). Psv-GFP cells were first observed 2 weeks after inoculation in xylem vessels close to the tumour (data not shown). At later times after inoculation (4 weeks) Psv-GFP cells were visualized either within the xylem vessels nearby the tumour (node) (Fig. 6B and E) or beyond the hyperplasic tissue (internode) (Fig. 6A and D). Finally, presence of strain PICF7 did not interfere with Psv movement outside the hyperplasic region and throughout the vascular system since the pathogen was also found in stems of Psv/PICF7 co-inoculated plants 2 weeks after inoculation (Fig. 6C and F).

### *Pseudomonas fluorescens* PICF7 decreases *Pseudomonas savastanoi*-induced necrosis of olive knots in woody olive plants

To finally check whether the inoculation of PICF7 induced the same effects in lignified olive plants than those observed on *in vitro* micropropagated explants, bioassays using pot-acclimated olive plants of two ages (1- and 2-year-old) and with lignified stems were carried out. Results showed that Psv/PICF7 co-inoculation did not significantly suppress olive knot disease onset and development in these plants. No significant differences (*P* > 0.05) were observed between plants treated or not with the BCA in volume, weight and density of analysed tumours in both experiments (Fig. S2). However, a trend towards an average decrease in volume and fresh weight was observed for Psv/PICF7 co-inoculated olive plants of both ages (Fig. S2). In addition, transverse section of knots induced by Psv/PICF7-treated plants showed a reduced necrosis of the internal tissues in comparison to control knots induced in plants inoculated with Psv alone, suggesting a delay in the maturation process of knots induced by the presence of the BCA. This effect was more evident in 2-year-old (Fig. S3) than in 1-year-old (data not shown) plants, probably due to the higher susceptibility to Psv infection of younger plants (Penyalver *et al*., 2006; Pérez-Martínez *et al*., 2010).

## Discussion

Successful management of olive knot disease is a complicated undertaking. The most frequent control measure (i.e. continuous application of copper-based bactericides), entails undesirable effects such as high costs, phytotoxicity and increasing risk of pathogen resistance (Krid *et al*., 2012). On the other hand, breeding for resistance has no current way of successful implementation since Psv-resistant cultivars are not yet available (Penyalver *et al*., 2006). Therefore, the use of BCAs appears as a promising control tool overcoming the adverse effects of chemical treatments and fitting modern sustainable agriculture criteria. Biological control of olive knot disease has been poorly explored, and only few studies have evaluated the effectiveness of diverse BCAs against Psv with variable results (Lavermicocca *et al*., 2002; Krid *et al*., 2012). The present study has examined the *in planta* interaction between *P. fluorescens* PICF7, an olive root endophyte effective against Verticillium wilt of olive (Mercado-Blanco *et al*., 2004; Prieto *et al*., 2009), and the causal agent of olive knot disease.

Results have proved that *P. fluorescens* PICF7: (i) inhibits the growth of Psv *in vitro* to a degree; (ii) colonizes and persists in stems of *in vitro-*propagated olive plants when artificially introduced; (iii) induces a transient decrease of Psv population on/in inoculated stem tissues; (iv) modifies the external macroscopic appearance of the tumours produced by the pathogen (less necrotic) on *in vitro*-propagated olive plants; (v) decreases the maturation process of Psv-induced tumours in woody olive plants (less internal necrosis); and (vi) alters the localization of the pathogen on/in tumours, that one being predominantly confined to internal regions of the knots. However, despite these consistent effects, PICF7 was not able to impair knot development under the experimental conditions here reported.

*Pseudomonas fluorescens* PICF7 is able to colonize and persist in both stems and roots of *in vitro*-propagated ‘Arbequina’ plants. Successful endophytic root colonization of *in vitro*-propagated olive explants by PICF7 has been corroborated even though plant material utilized in this present work differed in source, phenology and root system morphology than that used in a previous study (Prieto and Mercado-Blanco, 2008). Therefore, this BCA is able to endophytically colonize and persist in root tissues of different olive cultivars under diverse experimental conditions (Prieto *et al*., 2009; 2011; this study). On the other hand, this is the first study where an indigenous olive root inhabitant has been demonstrated to be successfully established in olive stem tissues after artificial inoculation, maintaining high population levels along time. Therefore, PICF7 can endure in different olive organs, opening new and interesting perspectives for its application as either preventive or palliative BCA in olive. Movement of PICF7 cells from roots to above-ground organs throughout the vascular system could not be faithfully assessed. Previous works using fluorescently tagged PICF7 discarded the presence of this bacterium neither within the xylem vessels of the roots nor in aerial tissues (Prieto and Mercado-Blanco, 2008; Prieto *et al*., 2011). Therefore, PICF7 cells occasionally found in stem tissue macerates could be explained by stem contamination during the bacterization process.

Strain PICF7 effectively antagonized Psv NCPPB 3335 *in vitro*, although nothing is known on what bacterial trait(s) could be responsible for such inhibitory effect. However, *in vivo* bioassays did not show an effective long-term control of Psv but a transitory drop of the pathogen population size (Figs 1 and 3). Nevertheless, a clear modification of the macroscopic appearance of developed tumours (Fig. 2), and a definitive alteration of the pathogen localization in knots (Figs 4 and 5) were observed when the BCA was present. Whether these phenomena are due to effective antibiosis mechanism(s) deployed by PICF7 *in planta* remain to be elucidated. The fall of pathogen population at early times after co-inoculation with the BCA was consistently observed in independent bioassays. This could be related to biosynthesis of inhibitory compounds by PICF7 affecting the pathogen, to a faster growth rate of the BCA compared with that of the pathogen, or to competition for space and nutrients inside olive tissues between the two bacteria. Regardless the mechanism involved, the transient drop of Psv population seemed to have a dramatic influence on the allocation of the pathogen on/in the tumour structure, a situation that could be observed either at macroscopic (Fig. 3) and microscopic (Figs 4 and 5) levels. Thus, whereas NCPPB 3335 predominantly colonized the surface of the tumour in the absence of the BCA (Fig. 5A, C and E), the former was predominantly restricted to inner cavities of the tumour (Fig. 5B, D and F). This shift in Psv localization may explain the different external appearance of knots when PICF7 was present (Fig. 2B and D). Moreover, despite the fact that PICF7 could not effectively control knot development, changes observed when the BCA was present may pose important epidemiological consequences. Quesada and colleagues (2010a) demonstrated dissemination of epiphytic Psv cells over short distances within olive orchards, a phenomenon perhaps related to transportation of the pathogen in aerosols (Young, 2004). Indeed, the release of pathogen cells through knot exudates, which could serve as new inoculum source, has been previously related to the localization of Psv cells at the knot surface (Rodríguez-Moreno *et al*., 2009). Therefore, presence of PICF7 reducing the number of Psv cells located at the surface of the tumour could imply a decrease in the dissemination of the pathogen through knot exudates.

Some beneficial *Pseudomonas* spp., native colonizers of the rhizosphere of diverse plants, can elicit a specific systemic defence response against pathogens in their host plants, a phenomenon known as induced systemic resistance (ISR) (Bakker *et al*., 2007; Mercado-Blanco and Bakker, 2007). Recent functional genomics studies have demonstrated that colonization by *P. fluorescens* PICF7 induced a broad set of defensive responses in olive root tissues. For instance, the establishment of the BCA on/in roots of ‘Arbequina’ plants produced the differential expression of genes involved in, among others processes, plant hormones and phenylpropanoids biosynthesis, pathogen-related proteins synthesis and several transcription factor involved in systemic defensive responses, including ISR (Schilirò *et al*., 2012). Considering this antecedent, the olive–Psv interaction offered an excellent study system to examine whether PICF7 could trigger an effective systemic defence response against olive knot disease, since the pathogen and the BCA can be applied spatially separated after artificial inoculation (Van Loon *et al*., 1998). Results showed that despite the fact that strain PICF7 effectively colonized the roots of *in vitro-*propagated plants, even endophytically, knot development on stems was not suppressed or altered, and populations of the pathogen in hyperplasic tissues were similar in both PICF7 root-bacterized and control plants. Therefore, while PICF7 is able to trigger a broad array of defensive responses in olive roots, including genes involved in ISR and systemic acquired resistance (SAR) responses (Schilirò *et al*., 2012), control of olive knot disease was not observed under experimental conditions assayed. A possible explanation would be that high virulence of the Psv strain used in this study (Rodríguez-Moreno *et al*., 2008) could overcome any potential systemic response from roots.

Invasion of newly formed xylem vessels inside olive knots induced by Psv strain NCPPB 3335 has been previously reported; however, pathogen cells could not be detected outside the knot area (Rodríguez-Moreno *et al*., 2009). An additional and interesting finding here performed was the demonstration that strain NCPPB 3335 is able to move from the inoculation site (and from developed tumours) through the xylem vessels. This phenomenon has been hardly evidenced before, particularly using a methodology that does not imply tissue fixation or staining procedure. Systemic invasion of oleander (*Nerium oleander* L.) plants through laticifers and, less frequently, through xylem vessels has been reported for *P. savastanoi* pv. nerii (Wilson and Magie, 1964). On the other hand, movement of Psv cells through the xylem vessels has been related to the formation of secondary knots in olive stems (Penyalver *et al*., 2006). Visualization of Psv cells within xylem cells in stained stem sections has been earlier reported (Marchi *et al*., 2009). However, the present study has clearly showed living GFP-tagged Psv cells directly visualized beyond the inoculation point.

In conclusion, by implementing powerful biotechnological and microscopy tools we have been able to uncover phenomena taking place during the interaction between a native bacterial endophyte of olive roots and a pathogen naturally occurring in above-ground tissues of the same plant host. This basic knowledge may have interesting practical information from an epidemiological point of view. Thus, although *P. fluorescens* PICF7 was not able to control olive knot disease, this BCA was demonstrated to colonize and establish in aerial olive tissues and to modify the colonization behaviour of the pathogen in tumours which, in addition, developed abnormally. Findings here reported can also be of interest to unravel the complex interplay that this pathogen could maintain with the microbiological consortia residing within olive knots (Hosni *et al*., 2011). Finally, Psv was undoubtedly and *in vivo* visualized migrating from the tumours to areas far beyond the hyperplasic regions, using the xylem vessels to do so.

## Experimental procedures

### Bacterial strains, growth conditions and inocula production

*Pseudomonas fluorescens* strain PICF7 (Mercado-Blanco *et al*., 2004), Psv strain NCPPB 3335 (Pérez-Martínez *et al*., 2007) and their fluorescently tagged derivatives – PICF7 carrying plasmid pMP4662 (Bloemberg *et al*., 2000), which harbours the red fluorescent protein (RFP) marker (Prieto and Mercado-Blanco, 2008), and NCPPB 3335 transformed with the plasmid pLRM1 harbouring the green fluorescent protein (GFP) marker (Rodríguez-Moreno *et al*., 2009), were used in this study. Growth conditions for *P. fluorescens* and Psv strains, and assessment of the stability of plasmids pMP4662 and pLRM1 in their respective hosts, have been earlier described by Prieto and Mercado-Blanco (2008) and Rodríguez-Moreno and colleagues (2009) respectively.

Bacterial inocula were prepared from cultures previously grown on KBA (King *et al*., 1954) or LBA (Miller, 1972) plates at 25–28°C for 24 h. Bacterial cells were resuspended in 10 mM MgSO_4_·7H_2_O by scraping bacterial lawns off with a sterile rod, washed twice (4500 r.p.m., 10 min) and resuspended in sterile 10 mM MgSO_4_·7H_2_O. Bacterial cell densities required for each experiment were established spectrophotometrically (A_600 nm_) by building up standard curves and culturing viable cells from serial dilution series onto KBA or LBA media (wild type) or KBA supplemented with the antibiotics tetracycline (20 mg l^−1^) (PICF7-RFP) or gentamicin (10 mg l^−1^) (NCPPB 3335-GFP).

### Plant material and plant growth conditions: *in vitro* propagated and lignified, pot-acclimated plants

Olive plants were micropropagated and rooted in Driver–Kuniyuki walnut (DKW) medium (Driver and Kuniyuki, 1984) from an *in vitro* germinated seed originated from a cv. ‘Arbequina’ plant (Rodríguez-Moreno *et al*., 2008) at the Instituto de Formación Agraria y Pesquera de Andalucía (IFAPA, Junta de Andalucía, Churriana, Málaga, Spain). Explants were transferred to sterile glass tubes with DKW without hormones and grew for at least 2 weeks in a growth chamber at 25 ± 1°C with a 16 h photoperiod. The length of plants used at the time of the bioassays was 80–100 mm long, with stems of 1–2 mm in diameter and always displaying 3–5 internodal segments.

For assays carried out with lignified plants (1 and 2 years old), plants were granted by the Instituto de Formación Agraria y Pesquera de Andalucía (IFAPA, Junta de Andalucía, Churriana, Málaga, Spain).

### *In vitro* antagonism of *P. savastanoi* NCPPB 3335 by *P. fluorescens* PICF7

To demonstrate whether *P. fluorescens* PICF7 exhibits *in vitro* antagonism against Psv, four drops (5 μl, 10^8^ cfu ml^−1^) of strain PICF7 were placed on the surface of KBA, LBA and PDA media plates previously inoculated with 100 μl of bacterial suspensions of strain NCPPB 3335 (ranging from 10^5^ to 10^8^ cfu ml^−1^). Two series of plates (two per assayed media) were incubated at 25 and 28°C, respectively, and after 72 h growth inhibition halos around PICF7 colonies were scored. Relative size of inhibition haloes was calculated according to the formula [halo diameter − colony diameter]/halo diameter. The experiments were carried out twice.

### Assessment of the colonization ability of *Pseudomonas fluorescens* PICF7 on/in tissues of *in vitro*-propagated Arbequina plants

To determine whether *P. fluorescens* PICF7 colonizes and persists on/in roots of *in vitro-*propagated Arbequina explants, 36 plants in total were uprooted from the DKW medium and their root systems dipped in a *P. fluorescens* PICF7 cells suspension (3.8 × 10^8^ cfu ml^−1^) (24 plants) or 10 mM MgSO_4_·7H_2_O (control treatment) (12 plants) for 15 min (Mercado-Blanco *et al*., 2004). After that, bacterized and non-treated roots were placed on top of several sheets of sterile filter paper 3 min to remove the excess of bacteria suspension or 10 mM MgSO_4_·7H_2_O.

To assess colonization and persistence on/in stems, drops (2 μl) of a PICF7 cells suspension (8.3 × 10^7^ cfu ml^−1^) were applied to intentional wounds made after removing a petiole of one intermediate leaf per plant (24 plants per treatment) with a sterile scalpel (Pérez-Martínez *et al*., 2010). After treatment, plants were placed again into sterile glass tubes containing DKW medium.

PICF7 populations on/in plant tissues were determined throughout both experiments by sampling three plants at 0, 3, 5, 7, 15, 30, 40 and 60 DAI. Thus, three root samples (100 mg) and three stem fragments (spanning 1 cm above and below from the inoculation point) from six independent plants were crushed in 1 ml of 10 mM MgSO_4_·7H_2_O under sterile conditions.

In addition, to verify whether PICF7 cells translocate from artificially bacterized roots to the stems, 12 plants were processed as described above. Root systems were dipped in a PICF7 cells suspension (4.0 × 10^8^ cfu ml^−1^) for 15 min. Then, plants were placed into sterile wide-mouthed bottles containing water agar medium to avoid PICF7-contamination of stems. Roots and stems of each plant were separately analysed at 15, 30, 40 and 50 DAI. Thus, the root system of each plant was removed and the stem was divided into three segments (i.e. basal, intermediate and apical sections). Subsequently, roots and stem segments were crushed in 1 ml of 10 mM MgSO_4_·7H_2_O. Serial dilutions of tissue macerates were plated onto KBA and incubated at 25°C for 48 h. After that, PICF7 colonies were counted and bacterial populations were determined along time. This assay was performed twice.

Manipulation of plants during bacteria inoculation, sampling and cell counting procedures were always conducted under sterile conditions within a laminar air flow cabinet.

### Olive–*Pseudomonas fluorescens*–*Pseudomonas savastanoi in vitro* bioassays

Two different types of bioassays (I and II) were conducted to investigate whether *P. fluorescens* PICF7 control olive knot disease of *in vitro*-propagated Arbequina plants. On the one hand, *P. fluorescens* PICF7 and Psv NCPPB 3335 were applied separately in different tissues (roots and stems respectively) to explore the possibility that the BCA could elicit a defence systemic response in Arbequina plants against the pathogen (type I bioassays). On the other hand, the BCA and the pathogen were simultaneously inoculated (cells suspension mix) in intentionally produced wounds in the stems (type II bioassays).

For type I bioassays, 48 explants (24 per treatment) were carefully uprooted from the growth media and dipped for 15 min in a bacterial suspension of strain PICF7 (3.8 × 10^8^ cfu ml^−1^) (BCA treatment) or 10 mM MgSO_4_·7H_2_O (control treatment), according to Mercado-Blanco and colleagues (2004). All plant manipulations were performed as indicated above except that, to avoid accidental contamination of stems with the BCA after root dip inoculation, bacterized plants were gently introduced in wide-mouthed bottles containing sterile water-agar (7 g agar l^−1^ distilled water) where they remained for the rest of the bioassay. One week after PICF7 treatment, stems were wounded once by excision of an intermediate leaf with a sterile scalpel and, immediately, a drop (2 μl) of a Psv suspension (7.7 × 10^7^ cfu ml^−1^) was applied to the wound under sterile conditions (Pérez-Martínez *et al*., 2010). Population size of strain NCPPB 3335 was assessed along the experiment, sampling stem fragments spanning 1 cm above and below the pathogen inoculation point at 0, 3, 5, 7, 15, 30, 40 and 60 DAI in control and PICF7-treated plants. Tissue manipulation was performed as indicated above. In addition, population of *P. fluorescens* PICF7 was also monitored in bacterized roots at the same time-points. For that purpose, root tissue samples (100 mg) were sampled and manipulated as previously indicated. Three independent plants were examined at each sampling time-point. Bacteria counts were performed as indicated for colonization assays (see above). The assay was performed twice.

In type II bioassays a drop (2 μl) of a bacterial suspension containing a mixture of *P. fluorescens* PICF7 (6.8 × 10^7^ cfu ml^−1^) and Psv NCPPB 3335 (2.86 × 10^7^ cfu ml^−1^) were applied to intentional wounds made on the stems of 24 *in vitro*-propagated ‘Arbequina’ plants as previously indicated (see above). A group of 24 additional plants were inoculated only with a suspension of Psv cells (9.4 × 10^7^ cfu ml^−1^) (control treatment). As in type I bioassays, population size of NCPPB 3335 was evaluated at 0, 3, 5, 7, 15, 30, 40 and 64 DAI in both control plants (only inoculated with NCPPB 3335) and plants co-inoculated with the BCA (PICF7) and the pathogen (NCPBB 3335). Population size of strain PICF7 was also score at the same time-points. Stem tissue segments (1 cm above and below the inoculation point) were crushed in 1 ml of 10 mM MgSO_4_·7H_2_O under sterile conditions. Serial dilutions of macerates were plated onto KBA and incubated at 25°C for 48 h as describe in colonization assay. This bioassay was performed four times.

Mean values of population size of Psv scored throughout experiments I and II in absence and presence of PICF7 were compare using Student's *t*-test (α = 0.05) along the experiments.

Development of knot disease symptoms on *in vitro* ‘Arbequina’ plants were captured with a digital camera (Panasonic FS 42, Lumix) and processed using PHOTOSHOP 4.0 software (Adobe Systems, San Jose, CA, USA).

For all bioassays involving *in vitro*-propagated ‘Arbequina’ plants, glass tubes or wide-mouthed recipients containing explants were always kept within controlled-growth chambers at 25 ± 1°C with a 16 h photoperiod and a light intensity of 65 μmol m^2^ s^−1^.

### Epifluorescence and confocal laser scanning microscopy

Epifluorescence and confocal laser scanning microscopy (CLSM), combined with fluorescent tagging of bacteria, were used to examine presence of the BCA, the pathogen and their potential interactions in *in vitro*-propagated ‘Arbequina’ plant tissues. Thus, in order to confirm changes in Psv NCPBB 3335 population levels in the presence of the BCA (see *Results* section), a specific bioassay was designed. Drops (2 μl) of suspensions of either GFP-tagged NCPPB 3335 alone (final cell density 4.8 × 10^7^ cfu ml^−1^) or a mix of GFP-tagged NCPPB 3335 (final cell density 1.3 × 10^8^ cfu ml^−1^) and strain PICF7 (final cell density 3.5 × 10^7^ cfu ml^−1^) were applied to intentionally made wound performed on the stems of *in vitro*-propagated plants as indicated above. Twelve plants per treatment were used. Development of knots was observed along 4 weeks after inoculation. Two plants were examined at 2, 3, 6, 8, 13 and 27 DAI using a stereoscopic fluorescence microscope (Leica MZ FLIII, Leica Microsystems, Wetzlar, Germany) equipped with a 100 W mercury lamp and a GFP2 filter (excitation, 480/40 nm). In addition, progress of knot symptoms was photographed with visible light using the same equipment. All images (epifluorescence and visible) were captured using a high-resolution digital camera (Nikon DXM 1200, Nikon Corporation, Tokyo, Japan) attached to the stereoscopic fluorescence microscope and processed using PHOTOSHOP 4.0 software (Adobe Systems).

Since knots generated on *in vitro*-propagated plants were found to differ at the macroscopic level (see *Results* section) depending on the presence or not of PICF7 cells in the inoculation mix, CLSM was used to examine at the microscopic level whether: (i) inner appearance of tumours may differ upon inoculation of Psv alone or Psv and PICF7 together; (ii) localization and/or distribution of NCPPB 3335 in the generated tumour cells may be influenced by the presence of PICF7; and (iii) Psv cells could be found beyond the inoculation point and the hyperplasic area and whether this potential pathogen spread may or not be influenced by the presence of the BCA. Tissue samples used for CLSM were obtained from ‘Arbequina’ *in vitro*-propagated plants inoculated with a suspension of NCPPB 3335-GFP cells (3.7 × 10^6^ cfu ml^−1^) or a mix containing NCPPB 3335-GFP (7.9 × 10^6^ cfu ml^−1^) and PICF7-RFP (3.0 × 10^7^ cfu ml^−1^) cells. Bacteria were inoculated according to the procedure previously described. Eight plants were used per treatment and two tumours were analysed each time-point. This bioassay was performed twice and sampling times were 4, 5, 7 and 9 weeks after inoculation for the first assay, and 1, 2, 4 and 6 weeks for the second one. *In vitro* ‘Arbequina’ plants were kept within controlled-growth chambers as indicated above. Transverse and longitudinal sections (40–60 μm thick) from stems at the inoculation site (initial stages of knot development) or from visible knots were obtained using a Vibratome Series 1000plus (TAAB Laboratories Equipment, Aldermarston, UK) as previously described (Prieto *et al*., 2007; Prieto and Mercado-Blanco, 2008). Tissue samples were always observed at the moment of sampling with an Axioskop 2 MOT microscope (Carl Zeiss, Jena GmbH, Germany) equipped with a krypton and an argon laser, controlled by Carl Zeiss Laser Scanning System LSM5 PASCAL software (Carl Zeiss). In addition, stem fragments containing the inoculation site (1 cm long) were sectioned longitudinally to assess the possible spread of Psv from the hyperplasic tissue, regardless the presence or absence of strain PICF7. Two plants per treatment were evaluated at 14, 30 and 42 DAI. GFP-tagged bacterial cells were excited with the 488 nm Argon laser line and were detected in the 500–520 nm window. RFP-tagged bacterial cells were excited with the 568 nm laser line and detected in the 580–620 nm window. Data were recorded and transferred for analysis to Zeiss LSM Image Browser version 4.0 (Carl Zeiss). Final figures were processed with PHOTOSHOP 4.0 software (Adobe Systems).

### Olive–*Pseudomonas fluorescens*–*Pseudomonas savastanoi* bioassays using lignified, pot-acclimated plants

Two bioassays (I and II) were carried out to determine whether coinoculation of strains Psv and PICF7 influenced the onset and/or development of knot disease in 1 and 2-year-old lignified ‘Arbequina’ plants, already acclimated in pots under greenhouse conditions. In bioassay I, three 2-year-old plants were wounded with a sterile scalpel at five sites along the main stem. Artificial wounds, 0.5 cm long, were made from the surface to the cambial area without removing the tab generated with the cut, and as described by Pérez-Martínez and colleagues (2007). Then, one drop (10 μl) of a bacterial suspension of PICF7 (6.5 × 10^8^ cfu ml^−1^), NCPPB 3335 (2.8 × 10^8^ cfu ml^−1^) or a mix of PICF7 (8.1 × 10^8^ cfu ml^−1^) and Psv (2.4 × 10^8^ cfu ml^−1^) were applied to each incision. Control plants were inoculated with 10 mM MgSO_4_·7H_2_O and three plants were used per treatment. In bioassay II, four 1-year-old plants per treatment were inoculated as describe above, although only three wounds per plant were generated in this case. As in experiment I, 10 μl drops were applied to the artificial wounds, containing bacterial suspensions of strain PICF7 (4.8 × 10^8^ cfu ml^−1^), Psv (4.1 × 10^8^ cfu ml^−1^) or a mix of PICF7 (5.0 × 10^8^ cfu ml^−1^) and Psv (3.3 × 10^8^ cfu ml^−1^). Control plants were inoculated with 10 mM MgSO_4_·7H_2_O.

Inoculated wounds were cover with their tabs, enveloped with parafilm and plants were bagged in order to increase relative humidity to 100%. Bacterized plants were kept within controlled-growth chambers at 25 ± 1°C with a 16 h photoperiod and a light intensity of 65 μmol m^2^ s^−1^. After 7 days plants were remove from bags. Along experiment measures of disease symptoms were recorded according to the scale: 0, no symptoms, to 10, the biggest developed tumour. Tumour necrosis was also held into account. Thus, (−), no necrosis; (+), moderate necrosis; and (++), severe necrosis were scored for each developed knot. At the end of the experiments (92 DAI) all tumours were photographed with a digital camera (Panasonic Fs 42 Lumix) and measures of knots fresh weight and volume, calculated by scoring their length, width and depth (Hosni *et al*., 2011), were recorded. To examine whether presence of PICF7 produced inner alterations in tumours, four knots per experiment (two per treatment) were sectioned by hand with a blade, photographed and macerated (1 g) in 2 ml of 10 mM MgSO_4_·7H_2_O. Serial dilutions of knots macerates were spotted on KBA plates supplemented with ampicilin (50 mg l^−1^), chloramphenicol (13 mg l^−1^) and cycloheximide (100 mg l^−1^) and KBA with nitrofurantoin (100 mg l^−1^) to score population of Psv.

## References

[b1] Bakker PAHM, Pieterse CMJ, Van Loon LC (2007). Induced systemic resistance by fluorescent *Pseudomonas* spp. Phytopathology.

[b2] Bardaji L, Pérez-Martínez I, Rodríguez-Moreno L, Rodríguez-Palenzuela P, Sundin GW, Ramos C, Murillo J (2011). Sequence and role in virulence of the three plasmid complement of the model tumor-inducing bacterium *Pseudomonas savastanoi* pv. savastanoi NCPPB 3335. PLoS ONE.

[b3] Bloemberg GV, Wijfes AHM, Lamers GEM, Stuurman N, Lugtenberg BJJ (2000). Simultaneous imaging of *Pseudomonas fluorescens* WCS365 populations expressing three different autofluorescent proteins in the rhizosphere: new perspectives for studying microbial communities. Mol Plant Microbe Interact.

[b4] Bull CT, De Boer SH, Denny TP, Firrao G, Fischer-Le Saux M, Saddler GS (2010). Comprehensive list of names of plant pathogenic bacteria, 1980-2007. J Plant Pathol.

[b5] Driver JA, Kuniyuki AH (1984). In vitro-propagation of Paradox walnut rootstock. Hortscience.

[b6] Ercolani GL (1978). *Pseudomonas savastanoi* and other bacteria colonizing surface of olive leaves in the field. J Gen Microbiol.

[b7] Gardan L, Bollet C, Abu Ghorrah M, Grimont F, Grimont PAD (1992). DNA relatedness among the pathovar strains of *Pseudomonas syringae* subsp. *savastanoi* Janse (1982) and proposal of *Pseudomonas savastanoi* sp. nov. Int J Syst Bacteriol.

[b8] Gardan L, Shafik H, Belouin S, Broch R, Grimont F, Grimont PAD (1999). DNA relatedness among the pathovars of *Pseudomonas syringae* and description of *Pseudomonas tremae* sp. nov. and *Pseudomonas cannabina* sp. nov. (ex Sutic and Dowson 1959). Int J Syst Bacteriol.

[b9] Hosni T, Moretti C, Devescovi G, Suarez-Moreno ZR, Fatmi MB, Guarnaccia C (2011). Sharing of quorum-sensing signals and role of interspecies communities in a bacterial plant disease. ISME J.

[b10] Kennelly MM, Cazorla FM, de Vicente A, Ramos C, Sundin GW (2007). *Pseudomonas syringae* diseases of fruit trees. Progress toward understanding and control. Plant Dis.

[b11] King EO, Ward MK, Raney DE (1954). Two simple media for the demonstration of pyocyanin and fluorescin. J Lab Clin Med.

[b12] Krid S, Rhouma A, Mogou I, Quesada JM, Nesme X, Gargouri A (2010). *Pseudomonas savastanoi* endophytic bacteria in olive tree knots and antagonistic potential of strains of *Pseudomonas fluorescens* and *Bacillus subtilis*. J Plant Pathol.

[b13] Krid S, Triki MA, Gargouri A, Rhouma A (2012). Biocontrol of olive knot disease by *Bacillus subtilis* isolated from olive leaves. Ann Microbiol.

[b14] Lavermicocca P, Lonigro SL, Valerio F, Evidente A, Visconti A (2002). Reduction of olive knot disease by a bacteriocin from *Pseudomonas syringae* pv. ciccaronei. Appl Environ Microbiol.

[b15] Li W, Estrada-de los Santos P, Matthijs S, Xie G-L, Busson R, Cornelis P (2011). Promysalin, a salicylate-containing *Pseudomonas putida* antibiotic, promotes surface colonization and selectively targets other *Pseudomonas*. Chem Biol.

[b16] López-Escudero FJ, Mercado-Blanco J (2011). Verticillium wilt of olive: a case study to implement an integrated strategy to control a soil-borne pathogen. Plant Soil.

[b17] Marchi G, Mori B, Pollacci P, Mencuccini M, Surico G (2009). Systemic spread of *Pseudomonas savastanoi* pv. savastanoi in olive explants. Plant Pathol.

[b18] Mercado-Blanco J, Bakker PAHM (2007). Interactions between plants and beneficial *Pseudomonas* spp.: exploiting bacterial traits for crop protection. Antonie Van Leeuwenhoek.

[b19] Mercado-Blanco J, Rodríguez-Jurado D, Hervás A, Jiménez-Díaz RM (2004). Supression of verticillium wilt in olive planting stocks by root-associated fluorescent *Pseudomonas* spp. Biol Control.

[b20] Miller JH (1972). Experiments in Molecular Genetics.

[b21] Penyalver R, García A, Ferrer A, Bertolini E, Quesada JM, Salcedo CI (2006). Factors affecting *Pseudomonas savastanoi* pv. savastanoi plant inoculations and their use for evaluation of olive cultivar susceptibility. Phytopathology.

[b23] Pérez-Martínez I, Rodríguez-Moreno L, Matas IM, Ramos C (2007). Strain selection and improvement of gene transfer for genetic manipulation of *Pseudomonas savastanoi* isolated from olive knots. Res Microbiol.

[b22] Pérez-Martínez I, Rodríguez-Moreno L, Lambertsen L, Matas IM, Murillo J, Tegli S (2010). Fate of a *Pseudomonas savastanoi* pv. savastanoi type III secretion system mutant in olive plants (*Olea europaea* L.). Appl Environ Microbiol.

[b24] Prieto P, Mercado-Blanco J (2008). Endophytic colonization of olive roots by the biocontrol strain *Pseudomonas fluorescens* PICF7. FEMS Microbiol Ecol.

[b25] Prieto P, Moore G, Shaw P (2007). Fluorescence *in situ* hybridisation (FISH) on vibratome sections of plant tissue. Nat Protoc.

[b26] Prieto P, Navarro-Raya C, Valverde-Corredor A, Amyotte SG, Dobinson KF, Mercado-Blanco J (2009). Colonization process of olive tissues by *Verticillium dahliae* and its in planta interaction with the biocontrol root endophyte *Pseudomonas fluorescens* PICF7. Microb Biotechnol.

[b27] Prieto P, Schilirò E, Maldonado-González M, Valderrama R, Barroso-Albarracín JB, Mercado-Blanco J (2011). Root hairs play a key role in the endophytic colonization of olive roots by *Pseudomonas* spp. with biocontrol activity. Microb Ecol.

[b28] Quesada JM, Garcia A, Bertolini E, Lopez MM, Penyalver R (2007). Recovery of *Pseudomonas savastanoi* pv. savastanoi from symptomless shoots of naturally infected olive trees. Int Microbiol.

[b29] Quesada JM, Penyalver R, Pérez-Panadés J, Salcedo CI, Carbonell EA, López MM (2010a). Dissemination of *Pseudomonas savastanoi* pv. savastanoi populations and subsequent appearance of olive knot disease. Plant Pathol.

[b30] Quesada JM, Penyalver R, Pérez-Panadés J, Salcedo CI, Carbonell EA, López MM (2010b). Comparison of chemical treatments for reducing epiphytic *Pseudomonas savastanoi* pv. savastanoi populations and for improving subsequent control of olive knot disease. Crop Prot.

[b31] Ramos C, Matas IM, Bardaji L, Aragón IM, Murillo J (2012). *Pseudomonas savastanoi* pv. savastanoi: some like it knot. Mol Plant Pathol.

[b32] Rodríguez-Moreno L, Barceló-Muñoz A, Ramos C (2008). In vitro analysis of the interaction of *Pseudomonas savastanoi* pvs. savastanoi and nerii with micropropagated olive plants. Phytopathology.

[b33] Rodríguez-Moreno L, Jiménez AJ, Ramos C (2009). Endopathogenic lifestyle of *Pseudomonas savastanoi* pv. savastanoi in olive knots. Microb Biotechnol.

[b34] Rodríguez-Palenzuela P, Matas I, Murillo J, López-Solanilla E, Bardaji L, Pérez-Martínez I (2010). Annotation and overview of the *Pseudomonas savastanoi* pv. savastanoi NCPPB 3335 draft genome reveals the virulence gene complement of a tumour-inducing pathogen of woody hosts. Environ Microbiol.

[b35] Rokni Zadeh H, Khavazi KI, Asgharzadeh A, Hosseini-Mazinani M, De Mot R (2008). Biocontrol of *Pseudomonas savastanoi,* causative agent of olive knot disease: antagonistic potential of non-pathogenic rhizosphere isolates of fluorescent *Pseudomonas*. Commun Agric Appl Biol Sci.

[b37] Schilirò E, Ferrara M, Nigro F, Mercado-Blanco J (2012). Genetic responses induced in olive roots upon colonization by the biocontrol endophytic bacterium *Pseudomonas fluorescens* PICF7. PLoS ONE.

[b36] Schroth MN, Osgood JW, Miller TD (1973). Quantitative assessment of the effect of the olive knot disease on olive yield and quality. Phytopathology.

[b38] Sisto A, Morea M, Zaccaro F, Palumbo G, Iacobellis NS (1999). Isolation and characterization of *Pseudomonas syringae* susp. *savastanoi* mutants defective in hypersensitive response elicitation and pathogenicity. J Phytopathol.

[b39] Teviotdale BL, Krueger WH (2004). Effects of timing of copper sprays, defoliation, rainfall, and inoculum concentration on incidence of olive knot disease. Plant Dis.

[b40] Van Loon LC, Bakker PAH, Pieterse CMJ (1998). Systemic resistance induced by rhizosphere bacteria. Annu Rev Phytopathol.

[b41] Vlassak K, Holm L, Duchateau L, Vanderleyden J, De Mot R (1992). Isolation and characterization of fluorescent *Pseudomonas* associated with the roots of rice and banana grown in Sri Lanka. Plant Soil.

[b42] Wilson EE, Magie AR (1964). Systemic invasion of the host plant by the tumor-inducing bacterium *Pseudomonas savastanoi*. Phytopathology.

[b43] Young JM (2004). Olive knot and its pathogens. Australas Plant Pathol.

[b44] Young JM (2010). Taxonomy of *Pseudomonas syringae*. J Plant Pathol.

